# Predictors of overweight and obesity in five to seven-year-old children in Germany: Results from cross-sectional studies

**DOI:** 10.1186/1471-2458-8-171

**Published:** 2008-05-21

**Authors:** Christian J Apfelbacher, Adrian Loerbroks, John Cairns, Heidrun Behrendt, Johannes Ring, Ursula Krämer

**Affiliations:** 1 The London School of Hygiene and Tropical Medicine (LSHTM), Keppel Street, London WC1E, UK; 2 University Hospital Heidelberg, Department of Clinical Social Medicine, Thibautstr. 3, 69115 Heidelberg, Germany; 3 Divison of Public Health and Primary Care, Brighton and Sussex Medical School, University of Brighton, Falmer BN1 9PH, UK; 4 Institute of Psychology, University of Heidelberg, Hauptstrasse 47-51, 69117 Heidelberg, Germany; 5 Division of Environmental Dermatology & Allergy GSF/TUM, ZAUM (Centre for Allergy and Environment), Technical University Munich, Biedersteinerstraße 29, 80802 Munich, Germany; 6 Department of Dermatology and Allergy Biederstein, Technical University Munich, Biedersteiner Straße 29, 80802 Munich, Germany; 7 Institut für Umweltmedizinische Forschung at the Heinrich-Heine-University Düsseldorf, Auf'm Hennekamp 50, 40225 Düsseldorf, Germany

## Abstract

**Background:**

Childhood obesity is a serious public health problem and epidemiological studies are important to identify predictive factors. It is the aim of this study to analyse factors associated with overweight/obesity in samples of German children.

**Methods:**

35,434 five to seven year-old children (50.9% boys) participated in cross-sectional studies between 1991 and 2000 in several rural and urban areas in East and West Germany. Weight and height were measured and body mass index was calculated. International cut-off points, recommended by the International Obesity Task Force, were used to classify childhood overweight and obesity.

Predictive modelling was employed to analyse independently associated factors, using logistic regression to adjust for confounding.

**Results:**

15.5% were overweight, and 4.3% were obese. Female sex, other than German nationality, smoking in the living place and increasing birth weight were found to increase the odds of overweight and obesity, while increasing educational level, living space > 75 m^2 ^and breastfeeding for more than three months were inversely associated.

**Conclusion:**

The findings add to the evidence informing public health action, both through health promotion strategies (promoting breastfeeding, tackling smoking) and wider societal change management (addressing children from migrant families and families with low educational level).

## Background

Recent evidence from the German Health Interview and Examination Survey for Children and Adolescents (KiGGS) provides nationally representative data with respect to overweight and obesity in children and adolescents (three to 17 years old) for the first time [1]. 15% were classified as overweight and 6.3% were classified obese (exceeding the 90^th ^and 97^th ^percentile of the German reference data, respectively [2]).

Obesity cannot only result in many adverse health outcomes such as respiratory [3], cardiovascular [4] or metabolic complications [5] as well as body dissatisfaction [6], but has itself a multifactorial aetiology. Thus, previous studies examined a large number of potential predictors of childhood overweight and obesity.

Apart from demonstrating a substantial burden of overweight and obesity in children and adolescents, the KIGGS study showed overweight or obesity to be associated with lower socioeconomic status, migration background and the mother being overweight. Other studies in samples of German children have identified further factors associated with a lower prevalence of overweight/obesity, e.g. high parental education [7-11], and factors consistently associated with an increased prevalence, such as maternal smoking during pregnancy [7,9,12,13]. A review of international studies found socio-economic status to be inversely associated with overweight/obesity [14]. Ethnicity or nationality have also been linked in the patterning of overweight and obesity[15,16]. Little physical activity and watching TV have been implied in the rising prevalence of childhood overweight/obesity [16-18], as well as the consumption of energy-dense foods[16]. Meta-analyses have shown a protective effect of breastfeeding on the risk of overweight/obesity [19,20].

The cited studies used various different cut-off points to categorise overweight/obesity. Most studies used population-based cut-offs depending on the observed distribution at a given percentile [7,9-13,21] and only a few studies [8,22] used the cut-offs suggested by Cole et al. [23]. Cole and co-authors have developed a definition of overweight and obesity based on pooled samples from several countries, providing age- and sex-specific cut-off points. These reference values are recommended for international comparison [24].

We have previously reported prevalence data for large samples of five to seven-year-old German children (N = 35,434), using the cut-off points proposed by Cole and co-authors [25]. We found significantly increasing prevalences of overweight and obesity in both East and West Germany from 1991 to 2000. Overall, the prevalence of overweight was 15.5%, and the prevalence of obesity was 4.3%.

The aim of this paper is to investigate predictors of childhood overweight and obesity, drawing on the same data.

## Methods

### Study design

The study design has been described previously [25]. In brief, data was drawn from the study in school beginners in East and West Germany (SAWO) [26,27], and from the MIRIAM study (Multicenter International Study On Risk Assessment Of Indoor and Outdoor Air Pollution and Eczema Morbidity) [28]. Repeated cross-sectional studies were conducted in several German cities: Borken, Essen, Duisburg, Köln, and Augsburg (MIRIAM) in the West of Germany; Leipzig, Halle, Magdeburg, Merseburg (from 1994), and four small rural towns in the Altmark (Salzwedel, Gardelegen, Osterburg and Kloetze) in the East of Germany.

All boys and girls entering primary school between 1991 and 2000 at one of the study sites were eligible to participate. Written consent was obtained from the children's parents. Every three years (1991, 1994, 1997, 2000), questionnaires were sent to the children's parents in the West, with the exception of Augsburg (1996, 2000). In the East, questionnaires were sent each year. Weight and height were measured at the obligatory school entry examinations which were held at the local health authorities. Height was measured without shoes and weight was measured in underwear.

Ethics approval was obtained from the ethics committees of the medical associations in Bavaria and Saxony-Anhalt, Germany for the original studies.

### Outcome variables: overweight and obesity

Body mass index (BMI) was calculated as weight/(height)^2 ^[kg/m^2^]. Our analysis is restricted to five to seven-year-old children. The cut-off points as suggested by Cole et al. [23] increase with age (per additional 6 months of age) and are generally higher for boys than for girls.

### Exposure variables

Besides age and sex, the questionnaire contained information on socio-demographic characteristics, environmental exposures and early-life factors. The following variables were included:

#### Nationality of the child

A child was classified as German if at least one parent reported German nationality. Until 1994 (East) and 1993 (West) the child's nationality was asked directly.

#### Education

Parents were asked about their total years of education and the education of their current partner if applicable. Parental educational level (based on the parent with the most total years of schooling) was classified as less than 10 years, 10 years and more than 10 years.

#### Living space > 75 m^2^

Parents were asked how many square metres (m^2^) the child's living space had. The median size was 75 m^2 ^and a binary variable with values <= 75 m^2 ^and > 75 m^2 ^was derived.

#### Number of persons living in the child's living place

Parents were asked how many persons were living in the child's living place. The variable used for analysis had the following values: 1, 2, 3, 4, 5, 6, 7, 8, 9 where 9 indicates 9 and more persons.

#### Single child

Parents were asked how many older/younger siblings their child had. From this information, a variable "single child" was created.

#### Attendance of day nursery or being otherwise looked after

Parents were asked whether the child spent more than **six **hours per day at a place other than the living address. If so, it is assumed that the child attended a day nursery or was otherwise looked after.

#### Smoking in the living place

Parents were asked whether there was smoking in the place where the child was living at the time.

#### Passive smoking during the first three years of life

Parents were asked whether anybody smoked in the place where the child spent most of his/her time during the first three years of life.

#### Smoking during pregnancy

Parents were asked whether the mother of the child smoked during pregnancy.

#### Breastfeeding for more than three months

Parents were asked for how many weeks mothers breastfed their children. From this information, a variable "breastfeeding for more than three months" was derived.

#### Preterm delivery

Parents were asked whether the birth of their child was premature.

#### Birth weight

Parents were asked about the birth weight of their children. The continuous variable was then transformed into the following categories: < 2500 g; 2500 g – < 3000 g; 3000 g – < 3500 g; 3500 g – < 4000 g; >= 4000 g.

#### Year of study

1991, 1992, 1993, 1994, 1995, 1996, 1997, 1998, 1999, 2000.

#### Study region

The geographically defined areas were coded according to whether they were located in the East or in the West of Germany.

#### Study location

The geographically defined areas were coded according to whether they were located in a rural or an urban area.

### Statistical analysis

Data analysis was performed with SAS^® ^Version 9.1 WIN. Subjects were included only if there were no missing data on age, sex and BMI. Prevalence differences between groups were tested using chi squared tests. Trends across categories were analysed using Cochran-Armitage trend tests. Logistic regression was performed to obtain adjusted odds ratios (ORs) and 95% confidence intervals (95% CIs), measuring the association between exposure variables and the odds of being overweight or obese, respectively, in univarate and in mutually adjusted models. Model fit was evaluated by performing Hosmer and Lemeshow Goodness-of-Fit tests, testing the association of predicted probabilities and observed responses [29].

## Results

The response rate for the questionnaire was 79% of those children who participated in the school entry examinations in the respective places and years. Table 1 shows the characteristics of the study population. The average BMI was 15.8 kg/m^2^. 15.5% of the participating children were classified as overweight and 4.3% as obese. The children's average age was 6.3 years with half of the study population being male. The large majority had a German nationality. Most children came from urban areas and from the Eastern study regions and had a birth weight between 2500 g and 4000 g. The level of parental education was comparably high with only 11.5% reporting less than 10 years of schooling. About half of the children lived in a place sized > 75 m^2^, about half attended day nursery and roughly every second child was exposed to environmental smoking at home. About one in three children was a single child, exposed to smoking during the first three years of life and was breastfed more than three months. Only a minority of children was delivered preterm or was exposed to maternal smoking during pregnancy.

**Table 1:** Characteristics of the study population (N = 35,434)

**Characteristic**	**Mean**	**SD**
Age [years]	6.28	0.34
Weight [kg]	22.85	4.11
Height [cm]	120.05	5.53
BMI [kg/m^2^]	15.77	2.03
No of persons in the child's living place (N = 34,997)	3.91	1.06

	**N**	**%**
Overweight	35,434	15.48
Obese	35,434	4.33
Male sex	35,434	50.89
Age group	35,434	
5.0 – < 5.5 years		0.23
5.5 – < 6.0 years		23.98
6.0 – < 6.5 years		47.91
6.5 – < 7.0 years		26.15
7.0 – < 7.5 years		1.42
7.5 – < 8.0 years		0.32
German nationality	35,146	92.22
Study region: East	35,434	67.73
Study location: Urban	35,434	76.14
Parental educational level:	33,984	
< 10 years of schooling		11.48
10 years of schooling		44.82
> 10 years of schooling		43.69
Living space > 75 m^2^	34,095	49.51
Single child	34,997	33.86
Attendance of day nursery	33,826	56.49
Smoking in the living place	33,208	45.00
Passive smoking during the first 3 years of life	34,693	31.95
Smoking during pregnancy	34,578	13.72
Breastfeeding > 3 months	33,106	31.63
Preterm delivery	34,540	6.45
Birth weight	34,809	
< 2500 g		5.76
2500 – < 3000		17.00
3000 – < 3500		38.32
3500 – < 4000		29.45
> 4000		9.47

### Univariate associations

In univariate analyses (Table 2), female sex, increasing age group, other than German nationality, living in an urban area, single child, smoking in the living place, passive smoking during the first three years of life, smoking during pregnancy and increasing birth weight were found to increase the odds of overweight. Living in the East of Germany, increasing parental educational level, living space > 75 m^2^, increasing number of persons living in the child's place, attendance of a day nursery, breastfeeding for more than three months and preterm delivery were found to be inversely associated with overweight. All exposure variables were significantly associated with overweight (p < 0.05). There was a significantly decreasing trend in prevalence with increasing parental education and increasing number of persons in the household. There was also a significantly increasing trend in prevalence with increasing birth weight category.

**Table 2:** Univariate associations of possibly predictive variables with overweight

**Variables**	**Prevalence of overweight (%)**	**P value**	**Odds Ratio [OR]**
Sex			
Male	13.77 (N = 18,032)	< 0.0001	Female vs Male 1.31 (1.23–1.38)
Female	17.25 (N = 17,402)		
Age group			
5.0 – < 5.5	17.50 (N = 80)	0.02	Per unit increase in age group: 1.04 (1.00 – 1.08)
5.5 – < 6.0	14.53 (N = 8,498)		
6.0 – < 6.5	15.77 (N = 16,977)		
6.5 – < 7.0	15.92 (N = 9,265)		Per unit increase in age: 1.12 (1.03 – 1.22)
7.0 – < 7.5	12.35 (N = 502)		
7.5 – < 8.0	18.75 (N = 112)		
Nationality			
German	14.85 (N = 32,413)	< 0.0001	Other than German vs German 1.68 (1.52 – 1.84)
Other than German	22.61 (N = 2,733)		
Study region			
East	14.34 (N = 23,998)	< 0.0001	East vs West 0.77 (0.72–0.82)
West	17.87 (N = 11,436)		
Study location			
Urban	15.86 (N = 26,981)	0.0004	Urban vs Rural 1.13 (1.06–1.22)
Rural	14.26 (N = 8,453)		
Parental educational level			
< 10 years of schooling	19.43 (N = 3,902)	< 0.0001***	Per unit increase in educational level 0.79 (0.75–0.82)
10 years of schooling	16.29 (N = 15,233)		0.81 (0.74–0.88)*
> 10 years of schooling	13.13 (N = 14,849)		0.63 (0.57–0.69)*
Living space			
<= 75 m^2^	16.74 (N = 17,213)	< 0.0001	>75 m^2 ^vs <= 75 m^2 ^0.82 (0.77 – 0.87)
> 75 m^2^	14.13 (N = 16,882)		
No. of persons in the child's living place		< 0.0001 ***	Per unit increase in no. of persons: 0.91 (0.88–0.94)
Single child			
No	14.42 (N = 23,148)	< 0.0001	Yes vs No: 1.26 (1.19 – 1.34)
Yes	17.49 (N = 11,849)		
Attendance of day nursery			
No	15.87 (N = 14,718)	0.02	Yes vs No: 0.93 (0.88 – 0.99)
Yes	14.98 (N = 19,108)		
Smoking in the living place			
No	14.14 (N = 18,266)	< 0.0001	Yes vs No: 1.28 (1.21–1.36)
Yes	17.45 (N = 14,942)		
Passive smoking during the first three years of life			
No	14.74 (N = 11,084)	< 0.0001	Yes vs No: 1.17 (1.10 – 1.25)
Yes	16.84 (N = 23,609)		
Smoking during pregnancy			
No	15.04 (N = 29,833)	< 0.0001	Yes vs No: 1.26 (1.16 – 1.36)
Yes	18.19 (N = 4,745)		
Breastfeeding for more than three months			
No	15.79 (N = 22,634)	< 0.0001	Yes vs No: 0.86 (0.81 – 0.92)
Yes	13.94 (N = 10,472)		
Preterm delivery			
No	15.63 (N = 32,313)	0.01	Yes vs No: 0.85 (0.75 – 0.96)
Yes	13.56 (N = 2,227)		
Birth weight			
< 2500 g	9.77 (N = 2,006)	< 0.0001***	Per unit increase in birth weight category 1.31 (1.27 – 1.35)
2500 – < 3000 g	11.92 (N = 5,916)		1.25 (1.06 – 1.48)**
3000 – < 3500 g	14.12 (N = 13,340)		1.52 (1.30 – 1.77)**
3500 – < 4000 g	17.58 (N = 10,251)		1.97 (1.69 – 2.30)**
> 4000 g	24.09 (N = 3,296)		2.93 (2.48 – 3.47)**

*compared to educational level I (< 10 years of schooling)

**compared to low birth weight (< 2500 g)

***Cochran-Armitage Trend Test

Univariate analyses for obesity (Table 3) revealed the same picture as the analyses for overweight with the exception that age group was not significantly related to obesity.

**Table 3:** Univariate association of predictive variables with obesity

**Variables**	**Prevalence of obesity (%)**	**P value**	**Odds Ratio [OR]**
Sex			
Male	4.01 (N = 18,032)	0.003	Female vs Male 1.17 (1.06–1.30)
Female	4.66 (N = 17,402)		
Age group			
5.0 – < 5.5	2.50 (N = 80)	0.18	Per unit increase in age group: 0.96 (0.90–1.03)
5.5 – < 6.0	4.55 (N = 8,498)		
6.0 – < 6.5	4.37 (N = 16,977)		
6.5 – < 7.0	4.04 (N = 9,265)		Per unit increase in age: 1.00 (0.86–1.16)
7.0 – < 7.5	3.98 (N = 502)		
7.5 – < 8.0	8.04 (N = 112)		
Nationality			
German	4.04 (N = 32,413)	< 0.0001	Other than German vs German 1.95 (1.68 – 2.28)
Other than German	7.61 (N = 2,733)		
Study region			
East	3.88 (N = 23,998)	< 0.0001	East vs West 0.73 (0.66–0.81)
West	5.26 (N = 11,436)		
Study location			
Urban	4.53	0.001	Urban vs Rural 1.24 (1.09–1.41)
Rural	3.69		
Parental educational level			
< 10 years of schooling	6.23 (N = 3,902)	< 0.0001 ***	Per unit increase in educational level 0.68 (0.63 – 0.73)
10 years of schooling	4.87 (N = 15,233)		0.77 (0.66 – 0.90)*
> 10 years of schooling	3.06 (N = 14,849)		0.48 (0.41 – 0.56)*
Living space			
<= 75 m^2^	4.92 (N = 17,213)		> 75 m^2 ^vs <= 75 m^2 ^0.75 (0.67–0.83)
> 75 m^2^	3.73 (N = 16,882)		
Number of persons in the child's living place		< 0.0001 ***	Per unit increase in no of persons: 0.90 (0.86–0.95)
Single child			
No	3.95 (N = 23,148)	< 0.0001	Yes vs No: 1.30 (1.17–1.45)
Yes	5.08 (N = 11,849)		
Attendance of day nursery			
No	4.59 (N = 14,718)	0.01	Yes vs No: 0.88 (0.79–0.98)
Yes	4.05 (N = 19,108)		
Smoking in the living place			
No	3.65 (N = 18,266)	< 0.0001	Yes vs No: 1.46 (1.32–1.63)
Yes	5.25 (N = 14,942)		
Passive smoking during the first three years of life			
No	3.92 (N = 23,609)	< 0.0001	Yes vs No: 1.33 (1.19 – 1.48)
Yes	5.14 (N = 11,084)		
Smoking during pregnancy			
No	4.06 (N = 29,833)	< 0.0001	Yes vs No: 1.47 (1.29 – 1.68)
Yes	5.86 (N = 4,745)		
Breastfeeding for more than three months			
No	4.48 (N = 22,634)	< 0.0001	Yes vs No: 0.76 (0.68 – 0.86)
Yes	3.46 (N = 10,472)		
Preterm delivery			
No	4.42 (N = 32,313)	< 0.001	Yes vs No: 0.64 (0.50 – 0.83)
yes	2.87 (N = 2,227)		
Birth weight			
< 2500 g	1.94 (N = 2,006)	< 0.0001***	Per unit increase in birth weight category 1.35 (1.28 – 1.42)
2500 – < 3000 g	3.26 (N = 5,916)		1.70 (1.20 – 2.41)**
3000 – < 3500 g	3.91 (N = 13,340)		2.05 (1.48 – 2.85)**
3500 – < 4000 g	4.88 (N = 10,251)		2.59 (1.86 – 3.59)**
> 4000 g	7.62 (N = 3,296)		4.16 (2.95 – 5.85)**

*compared to educational level I (< 10 years of schooling)

**compared to low birth weight (< 2500 g)

***Cochran-Armitage Trend Test

### Multivariable models for overweight and obesity

In multivariable analyses (Table 4), female sex, other than German nationality, being a single child, smoking in the child's living place, smoking during pregnancy, preterm delivery, increasing birth weight and increasing year of study were found to be independent risk factors for overweight. Of these factors, only female sex, other than German nationality, smoking in the living place and increasing birth weight were found to be independent risk factors for obesity. Parental educational level, living space > 75 m^2^, the number of persons living in the child's living place and breastfeeding for more than three months were found to be inversely associated with overweight. The same factors were inversely related to obesity. In addition, there was evidence for a protective association of attendance of day nursery with respect to obesity.

**Table 4:** Predictive model for overweight and obesity (N = 26,777) [all analyses are mutually adjusted for all the listed factors]

	Overweight OR (95% CI)	Obesity OR (95% CI)
Female Sex	1.45 (1.35–1.55)	1.30 (1.15–1.47)
Age	1.05 (0.95–1.17)	0.86 (0.72–1.04)
Other than German nationality	1.52 (1.39–1.84)	1.60 (1.26–2.03)
Study region (East/West)	0.96 (0.87–1.06)	1.02 (0.86–1.22)
Study location (urban/rural)	0.97 (0.90–1.06)	1.08 (0.92–1.26)
Study year	1.06 (1.05–1.08)	1.10 (1.07–1.12)
Educational level*	0.87 (0.82–0.92)	0.80 (0.73–0.89)
Living space > 75 m^2^	0.86 (0.80–0.93)	0.80 (0.69–0.92)
Number of persons	0.91 (0.86–0.96)	0.91 (0.83–1.00)
Single child	1.16 (1.04–1.29)	1.13 (0.93–1.37)
Attendance of day nursery	0.97 (0.90–1.05)	0.87 (0.76–1.01)
Smoking in the living place	1.32 (1.21–1.43)	1.46 (1.26–1.70)
Passive smoking during the first three years of life	1.02 (0.94–1.11)	1.13 (0.98–1.32)
Smoking during pregnancy	1.11 (1.00–1.23)	1.13 (0.95–1.36)
Breastfeeding for more than three months	0.88 (0.81–0.95)	0.82 (0.71–0.95)
Preterm delivery	1.53 (1.30–1.80)	1.16 (0.84–1.62)
Birth weight**	1.43 (1.38–1.49)	1.49 (1.39–1.59)
Association of predicted probabilities and observed responses (p-value***)	0.15	0.53

*OR for a unit increase in educational level

**OR for a unit increase in birth weight level

*** corresponding to Hosmer and Lemeshow Goodness-of-Fit Test

## Discussion

The findings of our multivariable analyses are largely in line with previous investigations of samples of German children of similar age [7-13,21,22].

The study provides further evidence of an inverse association between parental education and both overweight and obesity in children, as documented by the majority of previous recently published international studies [14]. A finding from the longitudinal 1958 British birth cohort was that social class at birth predicted adult obesity (aged 33 years) in men [30]. It may be speculated that parental educational level at the time of birth or in infancy might be a predictor of overweight/obesity in children, but our study used information on current educational level. However, a change in parental educational level after the birth of children seems unlikely.

In a review, Livingstone points out that the association between social factors and childhood obesity is not well understood, and that social factors might interact in different ways for different individuals and population groups [31]. For Germany, the studies are consistent in the sense that all studies including the one presented in this paper found an inverse association of a high level of parental education with overweight/obesity [7-11].

The inverse association with living space > 75 m^2 ^may be explained by protective effects of high social status, reflecting the fact that more living space becomes affordable with increasing income. More living space could also reflect a higher number of siblings, and the reduced odds could actually be due to not being a single child.

However, the inverse association was found in a model controlling for educational level, number of persons in the child's household, and being a single child.

Regarding overweight, the protective association of an increasing number of persons in the household could be seen in conjunction with the adverse effect of being a single child without siblings. Obviously, a social context – either through siblings or other persons in the household or both – confers some kind of protection with respect to overweight. Whether more persons in the household mirror a better socio-economic profile, or a closer monitoring of the child's health behaviour, e.g. with regard to eating habits or more opportunities to engage in physical activity with siblings, or whether the effect is mediated via psycho-social pathways remains speculative. The tentative protective association with attendance of day nursery or otherwise being looked after may likewise reflect social status or demonstrate the adverse effect of being neglected as a child on the development of overweight/obesity.

A study performed in the West German city of Aachen found that the prevalence of overweight was twice as high in children with other than German nationality as compared with native German children [21]. Multivariate analyses showed that most of the difference was explained by know risk factors such as maternal education, watching TV, snacking and consumption of sweets and soft drinks. From this finding, it was concluded that overweight prevention should address high-risk environments rather than high-risk ethnic groups. In contrast to this finding, a study in Swiss children found an independent association of non-swiss nationality and overweight [16]. Likewise, our multivariate analysis showed other than German nationality to be a strong independent risk factor for overweight and obesity, after adjustment for education. This may be due to the major limitation in the multivariate analyses presented which did not include data on dietary habits, physical activity or the time spent watching TV. Low levels of total [17] or vigorous physical activity [16,17] and TV watching [16,18] have been implied as risk factors for childhood overweight/obesity in earlier studies. If they had been measured and were associated with nationality in our study, these variables mighty possibly in part explain the observed relation between nationality and overweight/obesity. We were also unable to adjust for parental overweight and obesity which is a strong predictor for overweight and obesity in children [16,18,32-35]. In a study performed in Northwest Germany, the inverse gradient between parental school education and overweight was even more pronounced in children who had overweight parents [10]. However, in a prospective population-based family study in Eastern Finland, a family history of obesity did not seem to be a good predictor of BMI during childhood [36].

With regard to the role of breastfeeding, the study in East German children study found an inverse association with obesity, but not with overweight [8]. This protective effect was stronger if the children were exclusively breastfed. Although this study used the same cut-off points as we did, it should be noted that the study populations are not directly comparable with respect to the age range. The finding of inverse associations between breastfeeding and overweight/obesity confirms the finding of a recent meta-analysis [19] showing that breastfeeding reduced the odds of obesity in childhood (adjusted OR in the fixed model: 0.78 (95% CI 0.71–0.85)). Providing further evidence for a potential causal relation, another meta-analysis documented a dose-response relationship between duration of breastfeeding and risk of overweight [20]. However, a third meta-analysis investigating breastfeeding (as opposed to formula feeding) and BMI in adolescence or adulthood concluded that publication bias and confounding (e.g., by maternal BMI) cannot be ruled out [37].

Previous studies showed low birth weight to be inversely related to overweight and obesity [7] and higher birth weight to be positively associated [9,18,33]. However, some studies failed to observe associations between weight at birth and BMI levels in childhood [34,36]. A study from North Germany observed sex-specific associations: in boys, low birth weight was positively associated with obesity and in girls a high weight at birth appeared to be a significant obesity risk factor [35].

With respect to the smoking variables included in the analysis, there was consistent evidence for an increased prevalence of both overweight and obesity when there was smoking in the living place, while smoking during pregnancy was related to overweight, but not to obesity. In contrast to this, both studies performed in Bavarian children and focusing on maternal smoking during pregnancy found an increased risk for obesity, too [9,12]. While smoking during pregnancy, especially in early pregnancy, may result in neuroendocrine or other metabolic dysregulation [38], smoking in the living place is most likely a proxy measure for a set of lifestyles which in turn might be linked to carrying excess fat. As we did not have information on dietary patterns, we were unable to test a possible link between smoking in the living place and, for instance, the consumption of energy-dense foods.

Further limitations arise from other methodological issues. The measurement of children's weight and height was based on physical examination and can hence be considered a gold standard. However, although measurements procedures were standardised, inter-rater variability cannot be excluded. In the statistical analysis, multicollinearity could lead to biased effect sizes. A positive correlation of variables such as the smoking variables would result in an underestimation of the effect size in the multivariable models. As expected, the smoking variables do indeed show positive correlations (data not shown). The univariable effect sizes would therefore represent overestimations. It is evident that the true effect sizes lie somewhere between the univariable and multivariable estimates.

With respect to the study design, the cross-sectional nature of the investigation does not strictly allow to infer causality from the identified associations between exposures and overweight/obesity. However, reverse causality seems unlikely in most of the associations found (for instance overweight/obesity leading to parental smoking or to breastfeeding).

Although some exposures will have preceded the outcome (e.g., birth weight, breastfeeding, preterm delivery), they were measured retrospectively. This might have introduced recall bias. Also, exposure information might generally be distorted because it is based on self-reports. Social desirability may be an issue (for instance reported smoking during first three years of the children's life). However, to bias estimates in either direction, the accuracy of reports would need to be differential, i.e. dependent on the weight status of the child. This seems unlikely, as weight for height was not the primary outcome measure of the studies.

## Conclusion

The study represents the largest study on predictive factors for overweight and obesity in German children so far. With regard to overweight and obesity, the study provides further evidence of protective associations of breastfeeding (more than three months) and of having a certain size of living space (> 75 m^2^). Additionally, evidence is seen of an inverse association with educational level while female sex, other than German nationality, smoking in the living place and increasing birth weight category have been shown to be risk factors. Together with the evidence in the published literature, this analysis forms a basis upon which preventive measures could be developed. A British longitudinal cohort study found little evidence of incident cases of overweight/obesity emerging over adolescence, supporting the notion that efforts need to target children at a young age [15]. Health promotion strategies, for instance, to promote breastfeeding should complement wider societal change, particular with regards to the most vulnerable groups, i.e. children of migrants and children from families with low parental education.

## Competing interests

The authors declare that they have no competing interests.

## Authors' contributions

CJA suggested the idea for the article and wrote the manuscript, supervised by UK and JC. UK was principal investigator of the studies in East and West Germany. HB and JR were principal investigators of the study in Augsburg, Southern Germany. AL assisted in writing and reviewing the manuscript. All authors approved the final version of the manuscript.

## Pre-publication history

The pre-publication history for this paper can be accessed here:


